# Bone marrow mesenchymal stem cell-derived endothelial cells increase capillary density and accelerate angiogenesis in mouse hindlimb ischemia model

**DOI:** 10.1186/s13287-020-01710-x

**Published:** 2020-06-08

**Authors:** Ziping Yao, Huihui Liu, Min Yang, Yun Bai, Bihui Zhang, Chengen Wang, Ziguang Yan, Guochen Niu, Yinghua Zou, Yuan Li

**Affiliations:** 1 Department of Interventional Radiology and Vascular Surgery, Peking University First Hospital, No. 8 Xishiku Street, Beijing, 100034 China; 2 Department of Hematology, Peking University First Hospital, No. 8 Xishiku Street, Beijing, 100034 China; 3 Department of Cell Biology, School of Basic Medical Sciences, Peking University Health Science Center, Beijing, China; 4 Department of Minimally Invasive Tumor Therapies Center, National Center of Gerontology, Beijing Hospital, Beijing, China

**Keywords:** Mesenchymal stem cells, Endothelial cells, Hindlimb ischemia model, Angiogenesis, Engraftment

## Abstract

**Background:**

Mesenchymal stem cells (MSCs) can improve limb perfusion and increase vessel density in a murine model of hindlimb ischemia. But low engraftment rate of those cells limited their therapeutic effect. Endothelial cells (ECs) play an important role in neovascularization. And MSCs can differentiate into ECs in vitro. The aim of this study is to investigate if EC differentiation of MSCs in vitro before transplantation is effective in improving therapeutic outcomes in the treatment of ischemic disease in a murine ischemia animal model.

**Methods:**

MSCs were isolated from the bone marrow of EGFP-transgenic mice by density gradient centrifugation. The identity of the MSCs was determined by their cluster of differentiation (CD) marker profile by flow cytometry. Inducing medium containing a few cytokines was applied to induce the MSCs to differentiate into ECs. Endothelial differentiation was quantitatively evaluated using flow cytometry, quantitative real-time PCR (qRT-PCR), immunofluorescence, Matrigel tube formation assay, and Dil-labeled acetylated low-density lipoprotein uptake assay. Mouse hindlimb ischemia model was made by excision of the femoral artery. Uninduced EGFP+ MSCs, induced EGFP+ MSCs, and PBS were intramuscularly injected into the gastrocnemius following ischemia no later than 24 h after operation. Restoration of blood flow and muscle function was evaluated by laser Doppler perfusion imaging. Immunofluorescence was conducted to evaluate the engraftment of transplanted MSCs. Histological analysis was performed to evaluate blood vessel formation.

**Results:**

Induced EGFP+ MSCs expressed endothelial markers and exhibited tube formation capacity. Mice in the induced EGFP+ MSCs group had a better blood perfusion recovery, enhanced vessel densities, higher engraftment, and improved function of the ischemic limb than those in the uninduced EGFP+ MSCs or PBS groups.

**Conclusions:**

This study reveals that after short-term pre-treatment in the EC-inducing medium, induced MSCs acquire stronger vessel formation capability and enhanced angiogenic therapeutic effect in the murine hindlimb ischemia model.

## Background

Peripheral arterial disease (PAD) is a clinical manifestation of obstruction of vascular territories supplying the lower limbs caused by mainly atherosclerosis and less commonly by inflammation disorders and arteriopathies [1]. It is estimated that 200 million people worldwide have PAD, resulting in a big burden for healthcare and patients’ quality of life [2]. When the disease develops to the advanced stage called chronic limb-threatening ischemia (CLTI), it often leads to amputation as limited treatment options are available. Moreover, about 20–30% of patients with CLTI are ineligible for revascularization including surgical bypass and angioplasty because of severe calcification of the arteries [3]. In patients with severe atherosclerotic disease of the native arterial circulation, administration of cell populations that are capable to activate the angiogenic program may result in the formation of new vessels, hence improve or restore perfusion of the affected limb [4].

Mesenchymal stem cells (MSCs) are multipotent non-hematopoietic, fibroblast-like cells that can be isolated from various tissue sources including the bone marrow, adipose tissue, dental origin, placenta, umbilical cord blood [5]. MSCs hold great potential for regenerative medicine because of their ability of self-renewal, paracrine effects, and differentiation into tissue-specific cells such as osteoblasts, chondrocytes, and adipocytes [6]. Vascular smooth muscle cells (SMCs) and endothelial cells (ECs) are two major components of the vessel wall and are essential for blood vessel formation and function. It has been proved that MSCs can differentiate into ECs and SMCs in vitro in the presence of various growth factors [7, 8]. Due to its paracrine property and differentiation ability, MSCs have been considered as an effective option for PAD.

However, one main obstacle of MSC transplantation for clinical usage is the poor engraftment and poor survival rate. A study using bone marrow-derived MSCs (BM-MSCs) for the treatment of wounds in normal and diabetic mice yielded satisfying wound healing effect although engraftment at 28 days was 2.5%, which is extremely low [9].

ECs differentiated from human induced pluripotent stem cells can increase capillary density and improve perfusion in a mouse hindlimb ischemia model [10]. Human bone marrow-derived CD31+ cells after short-term culture in endothelial cell medium have higher cell engraftment and vessel formation in a mouse myocardial infarction model and hindlimb ischemia model than uncultured cells [11], suggesting that the therapeutic potential and survival rate of stem cells in vivo could be improved by pre-stimulation in vitro. BM-MSCs can differentiate into ECs in vitro [8]. However, the therapeutic potential of BM-MSC-derived ECs for the treatment of ischemic diseases has not been reported.

In this study, we induced the differentiation of BM-MSCs into ECs and characterized their gene expression and functional properties in vitro. We then examined the benefit of BM-MSC-derived ECs in improving the therapeutic outcomes using a murine hindlimb ischemia model, a disease model for human PAD.

## Materials and methods

### Mouse

The enhanced green fluorescent protein (EGFP) transgenic mice were of C57BL/6 background and were purchased from the Jackson Laboratory (NO. 006567). BALB/c mice were purchased from SPF Biotechnology (Beijing, China). All mice were bred in the animal breeding facilities at Peking University First Hospital under specific pathogen-free conditions, and all studies were approved by the Ethics Committee of Peking University First Hospital.

### Cell culture

The bone marrow-derived mesenchymal stem cells (BM-MSCs) were obtained from the EGFP-transgenic mice. Bone marrow aspirates were harvested from mice by flushing the tibias using syringes with 20-gauge needles. Freshly isolated bone marrow was suspended in a medium with Dulbecco’s modified Eagle’s medium (DMEM) (Invitrogen, Carlsbad, CA, USA) containing 2% fetal bovine serum (FBS) (Invitrogen, Carlsbad, CA, USA) and 1% penicillin/streptomycin (Invitrogen, Carlsbad, CA, USA) in a 37 °C-5% CO_2_ incubator. The culture medium was refreshed every 3 days, and non-adherent cells were removed. Cells were passaged at 80% confluence. BM-MSCs at each passage were analyzed for the surface markers using flow cytometry. All cells used in all experiments were from passages 1–2.

### Differentiation of MSCs into ECs

MSCs were cultured in the inducing medium containing 50 ng/mL VEGF (PeproTech, Rocky Hill, NJ, USA), 10 ng/mL bFGF (PeproTech, Rocky Hill, NJ, USA), 20 ng/mL IGF (PeproTech, Rocky Hill, NJ, USA), 5 ng/mL EGF (PeproTech, Rocky Hill, NJ, USA), ascorbic acid, heparin, and 2% FBS. The medium was changed every 2 days. The process of differentiation was closely monitored with cell morphology. To confirm the endothelial phenotype, the induced cells were analyzed for EC-specific markers using a series of experiments after 7 days of culture.

### Flow cytometry of uninduced MSCs and induced MSCs

The quality of MSCs was ensured by measuring EGFP-positive cells using flow cytometry. MSCs were stained with CD44-APC/Cy7 (Dakewe Biotech Co., Ltd.), CD34-Violet 421 (Dakewe Biotech Co., Ltd.), CD31-PE (Dakewe Biotech Co., Ltd.), and CD29-Percp (Dakewe Biotech Co., Ltd.). We identified MSCs as cells positive for CD29 and CD44, but negative for CD31 and CD34. After BM-MSCs were cultured in inducing medium for 7 days, induced MSCs were stained with CD31-PE to detect the expression of endothelia-specific marker.

### Dil-labeled acetylated low-density lipoprotein uptake assay

The induced MSCs grown to 80% confluency in 24-well plates were washed twice with a serum-free medium before 10 μg/mL Dil-labeled acetylated low-density lipoprotein (Dil-Ac-LDL) (Sigma Chemicals, St Louis, MO) was added. After being incubated at 37 °C and 5% CO_2_ for 4 h, the cell layer was washed in PBS twice, then cells were fixed in 4% paraformaldehyde for 30 min. The nuclei were stained with 4′,6-diaminido-2-phenylindol (DAPI) (Zsbio, Beijing, China) before cells were observed and imaged under a fluorescent microscope.

### Matrigel tube formation assay

Matrigel tube formation assay was conducted to demonstrate the angiogenic activity of the induced cells and uninduced cells in vitro. Fifty microliters of Matrigel (Corning, Acton, MA, USA) was added to 96-well plates at a horizontal level to allow even distribution. Plates were then incubated at 37 °C for 30 min. Induced and uninduced MSCs were harvested and seeded in triplicate into the 96-well plate at a concentration of 1 × 10^4^ cells/well. All procedures were conducted on the ice. After 6–10 h of incubation, representative fields were randomly photographed using microscopy.

### Quantitative real-time PCR

After BM-MSCs were cultured in inducing medium for 7 days, qRT-PCR was performed to test the expression of von Willebrand factor (vWF), vascular endothelial growth factor receptor-2 (VEGFR-2), and platelet and endothelial cell adhesion molecule 1(PECAM-1) to confirm the presence of differentiated ECs. In brief, total RNA was isolated from the induced cells and uninduced cells separately using TRIzol Reagent. Extracted RNA was then reverse-transcribed using the RevertAid first-strand cDNA Synthesis Kit (Thermo, Waltham, MA, USA). qRT-PCR was performed on an ABI Prism 7500 PCR system using the SYBR Green RT-PCR Kit (Life, Foster City, CA, USA) according to the manufacturer’s protocol to detect the expression of target genes. The primers used were listed as follows:

GAPDH: forward, 5′-TGCCCAGAACATCATCCCT-3′; reverse, 5′-ATGCCTGCTTCACCACCTT-3′

vWF: forward, 5′-CTTCTGTACGCCTCAGCTATG-3′; reverse, 5′-GCCGTTGTAATTCCCACACAAG-3′.

VEGFR2: forward, 5′-CTGGAGCCTACAAGTGCTCG-3′; reverse, 5′-GAGGTTTGAAATCGACCCTCG-3′.

PECAM-1: forward, 5′-CAAGGCCAAACAGAAACCCG-3′; reverse, 5′-GCCTTCCGTTCTCTTGGTGA-3′.

The PCR was performed under the following cycling conditions: denaturation at 28 °C for 10 min, annealing at 37 °C for 120 min, and extension at 85 °C for 5 min. Data were analyzed by the comparative threshold cycle (CT) method and normalized against GAPDH controls.

### Mouse ischemic hindlimb model and cell transplantation

Hindlimb ischemia model was performed in male BALB/c mice (age of 10–12 weeks). Mice were anesthetized with avertin (2.5%). The unilateral hindlimb ischemia model was prepared as follows: separate the femoral artery from the femoral vein and nerve at the proximal location near the groin, then separate the femoral artery from the femoral vein at the distal location close to the knee and occlude the vessel using double knots. Next, transect the segment of the femoral artery between the distal and proximal knots by using the sterile technique (see supplement). Mice (*n* = 15) were randomly divided into 3 groups: the uninduced MSC group, the induced MSC group, and the control group (PBS). No later than 24 h after the model operation, each animal was injected with 5 × 10^5^ uninduced MSCs in 50 μl of PBS, 5 × 10^5^ induced MSCs in 50 μl of PBS, or 50 μl PBS alone intramuscularly into 3 different sites of the gastrocnemius muscle in the medial thigh. The perfusion of the ischemic limb was examined on days 0, 3, 7, 14, 21, and 28 using laser Doppler perfusion imaging (LDPI) (Periflux 5001 Master, Perimed AB, Stockholm, Sweden). Blood perfusion was quantified using the perfusion rate (the rate of average LDPI index of the ischemic limb (right) to the non-ischemic hind limb (left)). The clinical outcome of ischemic hindlimb on day 28 was evaluated, which was defined as four progressive levels: limb salvage, nail blackening, foot necrosis, and limb loss. The ischemic thigh areas were removed 28 days post-transplantation for immunofluorescence assay.

### Immunofluorescence staining

Immunofluorescence staining was performed on induced MSCs and uninduced MSCs to detect the expression of EC markers (CD34 and vWF). After 7 days of culture in DMEM (uninduced group) or inducing medium (induced group), cells were seeded into 24-well chambers, washed with PBS, fixed with 4% paraformaldehyde for 15 min, and permeabilized with 0.5% Triton X-100 for 30 min. After 1 h of blocking with 3% bovine serum albumin (BSA), cells were incubated with rabbit anti-CD34 (1:200, Beyotime, Shanghai, China) and rabbit anti-vWF (1:200, Bioss, Beijing, China) antibodies in a humidified chamber at 4 °C overnight. Cells were washed using PBS for three times before incubation with AlexaFluor-594-labeled goat anti-rabbit secondary antibody (1:500, Abcam, Cambridge, UK) at room temperature for 1 h in the dark. Finally, cells were counterstained with DAPI (Zsbio, Beijing, China) in antifade reagent and imaged under a fluorescent microscope. The control samples consisted of cells without primary antibodies were used to assess the background fluorescence.

Thigh tissues were fixed with acetone. Immunofluorescent staining was performed using primary antibodies against CD31 (1:200, Beyotime, Shanghai, China), alpha-smooth muscle actin (α-SMA) (1:200, Abcam, Cambridge, UK), and secondary antibodies conjugated to Alexa594 (1:500, Abcam, Cambridge, UK) in the dark. The nuclei were stained with DAPI (Zsbio, Beijing, China). Immuno-stained slides were imaged by confocal microscopy.

### Immunohistochemical assay

We examined the effects of the uninduced and induced MSCs on the vessel density of capillaries and arterioles using immunohistochemical assay. The gastrocnemius muscles were transversely cut into 2 equal sections (proximal to distal) and embedded in 2 separate paraffin blocks. Paraffin-embedded tissues were cut into 5-μm-thick sections. The sections were deparaffinized, rehydrated, and were incubated with a rabbit anti-α-SMA monoclonal antibody (1:500, Abcam, Cambridge, UK) and a rabbit anti-CD31 monoclonal antibody (1:50, Abcam, Cambridge, UK) overnight at 4 °C in the humidified chamber. After washing with PBS, the sections were incubated with the horseradish peroxidase (HRP)-conjugated goat anti-rabbit IgG (1:50, Beyotime, Jiangsu, China) for 30 min at 37 °C in the humidified chamber. The DAB chromogenic reagent kit (Zsbio, Beijing, China) was used to colorate at room temperature. Vessels that stained positive for smooth muscle α-actin were identified as arterioles. Vessels that stained positive for CD31 and less than 9 μm in diameter were identified as capillaries. Vessels were counted on transverse sections from each hindlimb in a blinded manner in 5 randomly selected high-power fields at × 20 magnification. Vascular images were captured by using an inverted light microscope (Olympus) and were analyzed by using Image-Pro Plus (Media Cybernetics, Rockville, USA). Arteriole density and capillary density were expressed as the number of vessels per field (× 20). Muscle sections were also stained with hematoxylin and eosin for structural analysis and to identify the nuclei. The gastrocnemius muscle from a normal mouse was used as a positive control.

### Statistical analyses

Data were expressed as the mean ± SEM. Two-group comparisons were performed using the two-tailed unpaired Student *t* test. Multi-group comparisons were performed using ANOVA and the Mann-Whitney post hoc test to determine the statistical significance within and between groups. A *p* value < 0.05 was considered statistically significant. Analyses were performed using GraphPad Prism 8 and SAS V9.2.

## Results

### Differentiation of MSCs into ECs

**Fig. 1:**
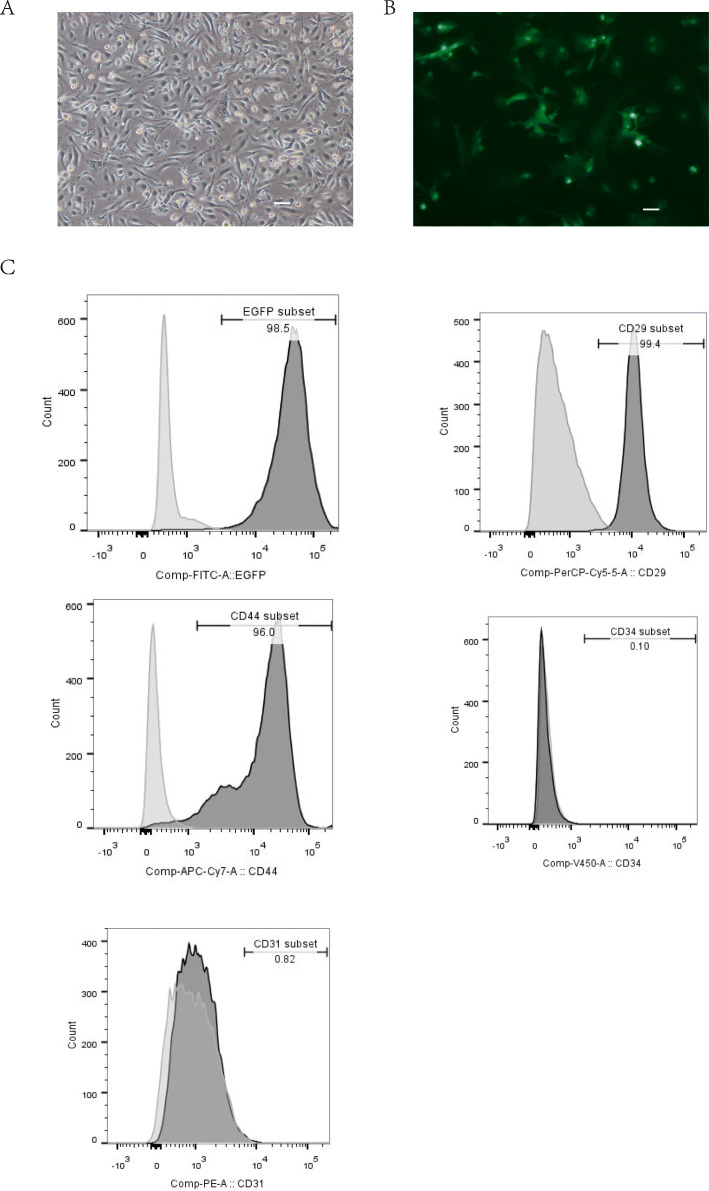
Characterization of BM-derived EGFP+ MSCs. **a** Morphological characteristics of EGFP+ MSCs. The cells showed a typical spindle-shaped morphology. Scale bar = 0.1 mm. **b** EGFP expression can be observed in most MSCs by fluorescence microscope. Scale bar = 0.1 mm. **c** Identification of EGFP+ MSCs by flow cytometry. As shown, MSCs are positive for EGFP (98.4%), CD29(99.4%), and CD44(96%) and negative for endothelial markers CD31(0.1%) and CD34 (0.82%)

MSCs isolated from the bone marrow showed a typical adherent spindle-like shape after about 5 to 6 days of culture in vitro (Fig. 1a). EGFP expression can be observed in cells under a fluorescence microscope (Fig. 1b). Flow cytometry confirmed 98.5% of these cells are EGFP positive. Also, these cells were positive for mesenchymal lineage markers of CD29 (99.4%) and CD44 (96%) while negative for the typical endothelial markers such as CD31 (0.1%) and CD34 (0.82%) (Fig. 1c).

**Fig. 2:**
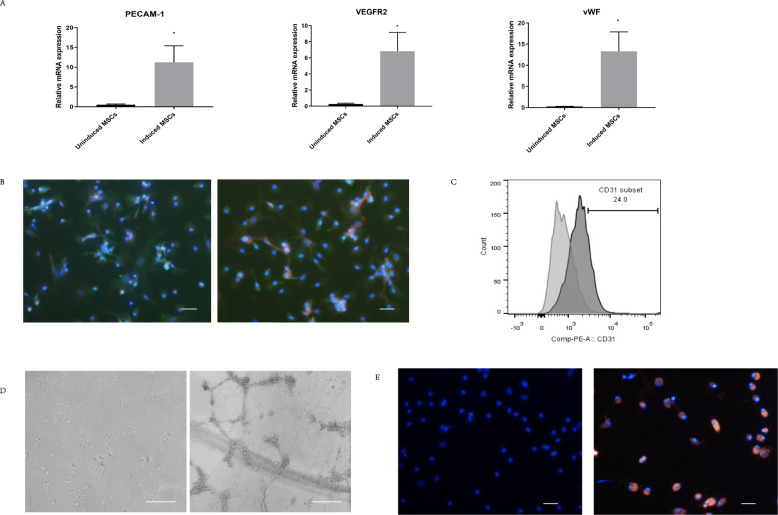
Characterization of induced MSCs. **a** Real-time PCR analysis: mRNA levels of vWF, PECAM-1, and VEGFR-2 were significantly higher in the induced MSCs compared with uninduced MSCs. Results are expressed as mean ± SEM. *p < 0.05, tested using Student *t* test. **b** Detection of endothelia-specific marker vWF and CD34 expression in the induced MSCs by immunofluorescence assay. Blue fluorescence signifies DAPI, red indicates CD34 or vWF, and green represents EGFP. Scale bar = 0.1 mm. **c** Flow cytometry showed increased CD31+ cell proportion after induction (24%). **d** Tube formation assay: induced MSCs form a capillary-like network on Matrigel after 6 h. Scale bar = 0.5 mm. **e** Induced MSCs take up acetylated LDL. Scale bar = 0.1 mm. Blue fluorescence signifies DAPI and red indicates DiI-labeled-acetylated LDL uptake in cells

After 7 days of culture in the inducing medium, we evaluated the differentiation status of the cells. Real-time PCR showed that mRNA transcript levels of EC markers such as vWF, PECAM-1, and VEGFR-2 were significantly increased in the induced cells. qRT-PCR revealed more than a 10-fold increase in the expression of VEGFR-2 (*p* < 0.05), and more than a 20-fold increase in the expression of vWF and PECAM-1 in the induced group (*p* < 0.05) (Fig. 2a). Immunofluorescence staining showed the expression of CD34 and vWF in induced cells, which are specific EC markers (Fig. 2b). Flow cytometry showed the percentage of CD31+ cells was 24% (Fig. 2c). Functional tests were further performed to validate the differentiated ECs. After 7 days of exposure to inducing medium, induced MSCs aligned and branched from the cell periphery to form tube-like structures (Fig. 2d). Vascular endothelial cells uptake lipoprotein through receptor-mediated endocytosis. We used DiI, a fluorescent probe, to label LDL to visualize the uptake of lipoproteins under fluorescence microscopy. Induced MSCs showed a high percentage of DiI-Ac-LDL^+^ cells (62%) (Fig. 2e). Thus, we have validated the differentiation of ECs through pre-treatment with an endothelial-inducing medium at the mRNA, protein, and cell function levels.

### Induced MSC transplantation improves blood perfusion in the ischemic hindlimb of BALB/C mice

**Fig. 3:**
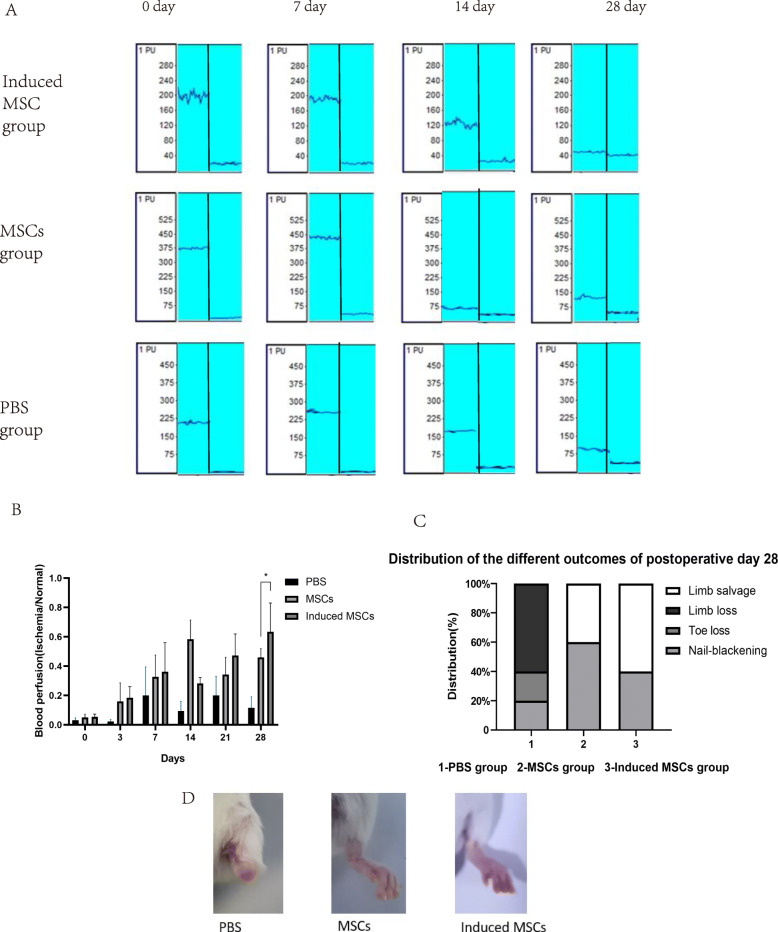
Evaluation of functional recovery in a murine hindlimb ischemia model. **a** The ratio of blood perfusion was investigated by laser Doppler perfusion imaging analysis in the ischemic limbs of normal mice injected with PBS, uninduced MSCs, and induced MSCs at 0, 7, 14, and 28 days post-operation. Representative pictures were shown. The former one is the normal control side, and the latter one is the operation side; data are expressed as the rate of LDPI index of the ischemic limb to the non-ischemic hind limb. **b** The ratio of blood perfusion was measured. Results are expressed as mean ± SEM; asterisk (*) indicates significant difference of the induced MSCs groups versus uninduced MSCs group, *p* < 0.05. **c** The distribution of the different outcomes on postoperative day 28. **d** Representative images illustrating the various outcomes: limb loss and limb salvage

The ability of induced MSCs and uninduced MSCs to induce or enhance blood perfusion in vivo was investigated using the hindlimb ischemia mouse model described in the “Materials and methods” section. Mice in the uninduced and induced MSCs group showed significantly higher blood perfusion than those in the PBS group. Mice in the uninduced MSCs group had similar or even better blood flow in the ischemic limb than those in the induced MSCs group in the second weeks (Fig. 3a, b). The blood perfusion of the uninduced MSCs group showed a sudden drop after day 14, suggesting an unstable blood recovery effect. The advantage of induced MSCs emerged later on day 21, and the effect continued to rise until the end of our observation (Fig. 3b). On day 28, the difference in blood perfusion among the groups was prominent, with the induced MSCs group showed much better perfusion recovery than the uninduced MSCs group. (*p* = 0.0431, Fig. 3b).

We then evaluate the levels of the recovery of ischemic hindlimb on day 28. Cell transplantation (uninduced and induced MSCs) groups raised the limb salvage rate (Fig. 3c). No mice showed final limb salvage in the PBS group: 3 (60%) had limb loss, 1 (20%) had toe loss, and 1 (20%) demonstrated nail blackening. Of the 5 mice that received uninduced MSC transplantation, 2 (40%) had limb salvage, while the other 3 (60%) had nail blackening. In the induced MSCs group, 3 mice (60%) recovered well with no loss or necrosis and 2 (40%) demonstrated nail blackening. A representative picture of each group was shown (Fig. 3d). This result is consistent with blood perfusion measured by LDPI in each group.

### Induced MSC transplantation improves arteriogenesis in the ischemic hindlimb of BALB/C mice

**Fig. 4:**
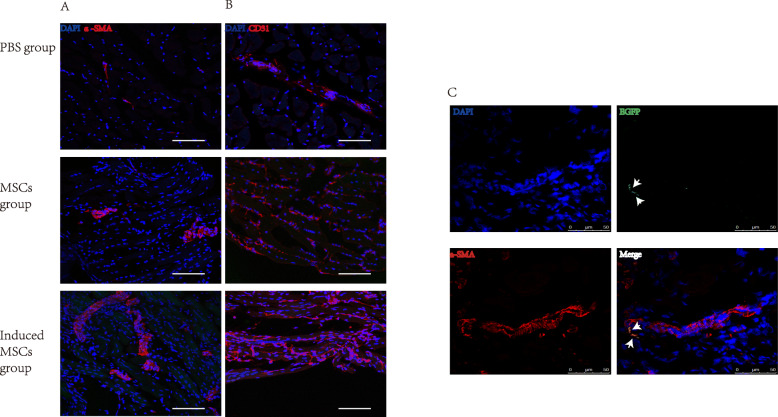
Effects of uninduced MSCs and induced MSCs on neovascularization at ischemic sites. On day 28 post-surgery, ischemia samples were analyzed to determine endothelial differentiation and neovascularization of transplanted MSCs at the injured sites. Vessel formation was confirmed by immunofluorescence staining for α-SMA (red) (**a**) and CD31(**b**). Scale bar = 75um. **c** In induced MSCs, GFP+ cells co-localized with α-SMA, indicating vasculature formation from transplanted cells. Scale bar = 50um

To explore the mechanism of induced MSCs in improving the blood perfusion, immunofluorescence staining was performed on ischemic injury tissues on day 28 to evaluate the neovascularization and engraftment. Compared to the mice in the uninduced MSCs group or PBS group, arteriole and capillary densities were significantly augmented in the ischemic tissues of mice that received induced MSC transplantation, indicated by the fluorescent density of the anti-α-SMA and anti-CD31 antibodies (Fig. 4a, b). Figure 4c illustrated the cell retention of induced EGFP+ MSCs 28 days after the transplantation. EGFP+ cells were found in the skeletal muscle in the areas of EGFP+-induced MSC injection, whereas no EGFP+ cells were found in the skeletal muscle that received EGFP+ uninduced MSC delivery, indicating an increase in cell engraftment or in vivo proliferation of EGFP+-induced MSCs. EGFP expression colocalized with α-SMA staining, suggesting transplanted cells integrated into the vasculature directly in ischemic limbs.

**Fig. 5:**
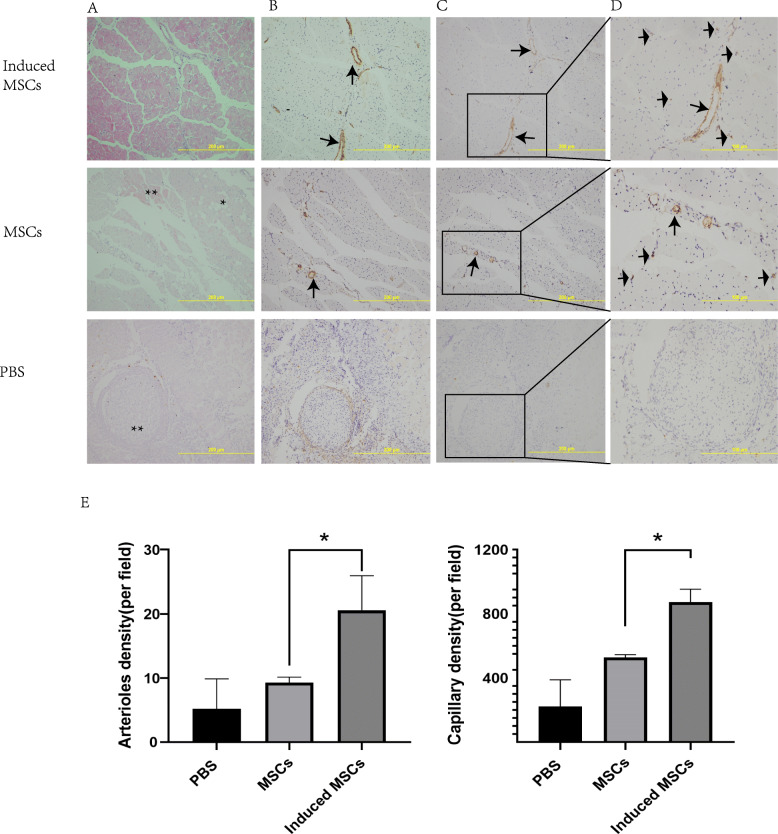
Histologic analysis of the limb muscles. Tissues were stained with **a** H&E staining to access necrotic (**), adipocyte infiltration (*), and normal issues; **b** α-SMA staining and **c** CD31 staining to access capillary (short arrow) and mature vessels (long arrow); and **d** CD31 staining under magnification × 40. **e** Standard qualification of the number of capillary and arterioles represented as the number of CD31-positive capillary per field (× 20) and the number of α-SMA-positive arterioles per field (× 20), respectively. Results are expressed as mean ± SEM; asterisk (*) indicates significant difference of the induced MSCs groups versus uninduced MSCs group, *p* < 0.05

Angiogenesis is essential for muscle recovery after ischemia as neovascularization provides nutrients and oxygen. To assess the muscle recovery, we performed histopathological staining to assess the degeneration and apoptosis of fibers by morphology (Fig. 5a). Compared to the PBS group, uninduced MSCs alleviated the degeneration and apoptosis of fibers to some extent, whereas induced MSCs significantly reduced the necrotic fibers and inflammatory cells in injured tissues. Furthermore, no major organ damage was observed, suggesting this injection is a safe treatment option.

To further identify capillaries and arterioles, we used antibodies α-SMA (Fig. 5b) and CD31 (Fig. 5c, d) to immune-stain tissue sections of ischemic limb 28 days after the transplantation treatment. The capillary density was markedly increased in mice that received induced MSCs (872.9 ± 79.35 vessels/field) compared with those in the uninduced MSCs group (527.1 ± 18.91 vessels/field) (*p* = 0.0167). Relative to the PBS group (5.20 ± 4.669 vessels/field) and the uninduced MSCs group (9.28 ± 0.855 vessels/field), induced MSC treatment showed the highest α-SMA+ arteriole density in the ischemic regions among all groups (20.56 ± 5.379 vessels/field) (*p* = 0.0036). These results suggested that transplantation of induced MSCs may restore ischemic tissues through increasing capillary and arteriolar densities.

## Discussion

Cell transplantation-based therapy for patients with CLTI is an important topic that has been under-investigated. Reduced oxygen supply and inflammation in the ischemic tissues often lead to the accumulation of reactive oxygen species, resulting in the death of transplanted cells [12]. To meet the oxygen and metabolic needs of ischemic tissues, enough functional vessels for sufficient perfusion are required. However, newly formed vessels generated by grafted cells are often immature and difficult to incorporate into host blood vessels even with adequate cell numbers [13]. The small number of mature vessels generated by grafted cells remains another unsolved problem [13]. Therefore, the need for a new cell type with higher tolerance against oxidative stress and stronger angiogenic potential is reinforced.

MSCs are promising in regenerative medicine because of their self-renewal and pluripotent abilities. Skewing MSCs to EC-like cells in vitro has been commonly achieved by using cytokines such as VEGF, EGF, FGF, and IGF. In our study, we successfully induced EC-like cells from BM-MSCs in the inducing medium. Flow cytometry showed the expression of EC surface marker CD31. The qRT-PCR and immunofluorescence staining confirmed the expression of EC markers vWF, CD34, VEGFR-2, and PECAM-1 at the mRNA and protein levels. These induced MSCs can form a tube-like structure and uptake LDLs, showing some functional properties of ECs.

Recent studies have demonstrated the possible enhancement of therapeutic efficacy in ischemic diseases by preconditioned graft cells [11]. Moreover, the EC-like cells induced in vitro from BM-MSCs already showed the ability of forming capillary-like structures in Matrigel. The induced MSCs also had upregulated expression of the VEGFR-2, which can be stimulated by VEGF, a major growth factor for angiogenesis. Whether preconditioning MSCs in the EC differentiation medium could improve its angiogenic effect in vivo is yet unknown. We tested the treatment effects of in vitro induced MSCs versus uninduced MSCs and PBS in a mouse ischemic hindlimb model, which was established to mimic the PAD in humans as it causes severe, stable, and uniform ischemia (the blood flow decreased at least 90% compared to normal limb). The BALB/c strain was selected for the ischemia model given its bad recovery and high necrosis rate in the limbs [14]. BM-MSCs have unique immunologic characteristics, such as low immunogenicity and immunoregulatory properties [15]. Previous study showed tolerance of infusing either autologous or donor-derived MSCs in unconditioned mice [16]. In our study, we did not observe obvious immune rejection of BM-MSCs of C57/BL6 background transplantation in the BALB/c mice.

In our study, we followed up the blood perfusion changes regularly after ischemic model surgery. The LDPI indicated better perfusion in mice that received either induced or uninduced MSCs than those received only PBS. This indicated that even uninduced MSCs showed some treatment benefits. This may be because that MSCs can differentiate into ECs in vivo after transplantation. However, the advantage of induced MSCs emerged in the later stage (2 weeks post-surgery). Induced MSC transplantation improved blood perfusion steadily and continuously. Blood flow began to restore 3 days after transplantation and steadily increased over time throughout the 28 days. In contrast, in mice that had uninduced MSC transplantation, the blood flow increased until day 14, followed a drop on day 21 and a mild increase on day 28 but not yet to the level on day 14. This was observed in almost every mouse in this group, indicating the fluctuant treatment effect of the uninduced MSCs. However, the mechanism of the phenomenon is unknown and worthy of further exploration.

One major obstacle of MSC utility for therapeutic angiogenesis is their low rate of engraftment in vivo as a consequence of the ischemic or inflammatory environment [17, 18]. This may explain the drop in the angiogenic effects of uninduced MSCs we observed in this study. Meanwhile, the EC-like cells, which was achieved by using specific cytokines in vitro, showed enhanced angiogenic effects and cell engraftment, which in turn further promoted blood flow recovery of the ischemic limb, which also explained the steady treatment effect of the induced MSCs. Indeed, the neovascularization in the ischemic tissues was confirmed by histological analysis. The immunohistochemistry with an antibody detecting endothelial marker CD31 to label the new vessels in the ischemic hindlimb and an antibody detecting marker of smooth muscle cells α-SMA showed increased capillary density and arteriole density of the induced MSCs group compared to the uninduced MCSs and PBS control groups. This result is consistent with LDPI data on day 28 post-implantation, thus further confirmed the efficacy of induced MSCs.

## Conclusion

This study provided evidences that in vitro MSC-derived EC-like cells acquired stronger capability in vessel formation and angiogenic effect in a mouse ischemic hindlimb model. Our study suggests that induced MSCs may be a promising therapeutic option for treating ischemic disease and worthy of further investigation.

## Supplementary information

**Additional file 1.** Induction of unilateral mouse hindlimb ischemia model.

**Additional file 2.** Supplementary figure.

## Data Availability

The authors confirm the availability of all data generated or analyzed in this manuscript.

## Abbreviations

MSC: Mesenchymal stem cell
EC: Endothelial cell
EGFP: Enhanced green fluorescent protein
CD: Cluster of differentiation
PAD: Peripheral arterial disease
CLTI: Chronic limb-threatening ischemia
SMC: Smooth muscle cell
BM-MSC: Bone marrow-derived MSCs
DAPI: 4′,6-Diaminido-2-phenylindol
vWF: von Willebrand factor
VEGFR-2: Vascular endothelial growth factor receptor-2
PECAM-1: Platelet and endothelial cell adhesion molecule 1
LDPI: Laser Doppler perfusion imaging
α-SMA: Alpha-smooth muscle actin

## References

[1] Kullo IJ, Rooke TW (2016). Clinical practice. Peripheral artery disease. N Engl J Med.

[2] Fowkes FG, Rudan D, Rudan I, Aboyans V, Denenberg JO, McDermott MM (2013). Comparison of global estimates of prevalence and risk factors for peripheral artery disease in 2000 and 2010: a systematic review and analysis. Lancet.

[3] Samura M, Hosoyama T, Takeuchi Y, Ueno K, Morikage N, Hamano K (2017). Therapeutic strategies for cell-based neovascularization in critical limb ischemia. J Transl Med.

[4] Frangogiannis NG (2018). Cell therapy for peripheral artery disease. Curr Opin Pharmacol.

[5] Samsonraj RM, Raghunath M, Nurcombe V, Hui JH, van Wijnen AJ, Cool SM (2017). Concise review: multifaceted characterization of human mesenchymal stem cells for use in regenerative medicine. Stem Cells Transl Med.

[6] Bronckaers A, Hilkens P, Martens W, Gervois P, Ratajczak J, Struys T (2014). Mesenchymal stem/stromal cells as a pharmacological and therapeutic approach to accelerate angiogenesis. Pharmacol Ther.

[7] Zhang X, Bendeck MP, Simmons CA, Santerre JP (2017). Deriving vascular smooth muscle cells from mesenchymal stromal cells: evolving differentiation strategies and current understanding of their mechanisms. Biomaterials.

[8] Wang C, Li Y, Yang M, Zou Y, Liu H, Liang Z (2018). Efficient differentiation of bone marrow mesenchymal stem cells into endothelial cells in vitro. Eur J Vasc Endovasc Surg.

[9] Wu Y, Chen L, Scott PG, Tredget EE (2007). Mesenchymal stem cells enhance wound healing through differentiation and angiogenesis. Stem Cells.

[10] Rufaihah AJ, Huang NF, Jame S, Lee JC, Nguyen HN, Byers B (2011). Endothelial cells derived from human iPSCS increase capillary density and improve perfusion in a mouse model of peripheral arterial disease. Arterioscler Thromb Vasc Biol.

[11] Kim SW, Houge M, Brown M, Davis ME, Yoon YS (2014). Cultured human bone marrow-derived CD31(+) cells are effective for cardiac and vascular repair through enhanced angiogenic, adhesion, and anti-inflammatory effects. J Am Coll Cardiol.

[12] Ott M, Gogvadze V, Orrenius S, Zhivotovsky B (2007). Mitochondria, oxidative stress and cell death. Apoptosis.

[13] Samura M, Morikage N, Suehiro K, Tanaka Y, Nakamura T, Nishimoto A (2016). Combinatorial treatment with apelin-13 enhances the therapeutic efficacy of a preconditioned cell-based therapy for peripheral ischemia. Sci Rep.

[14] Masaki I, Yonemitsu Y, Yamashita A, Sata S, Tanii M, Komori K (2002). Angiogenic gene therapy for experimental critical limb ischemia: acceleration of limb loss by overexpression of vascular endothelial growth factor 165 but not of fibroblast growth factor-2. Circ Res.

[15] Crop M, Baan C, Weimar W, Hoogduijn M (2009). Potential of mesenchymal stem cells as immune therapy in solid-organ transplantation. Transpl Int.

[16] Casiraghi F, Azzollini N, Cassis P, Imberti B, Morigi M, Cugini D (2008). Pretransplant infusion of mesenchymal stem cells prolongs the survival of a semiallogeneic heart transplant through the generation of regulatory T cells. J Immunol.

[17] Laflamme MA, Chen KY, Naumova AV, Muskheli V, Fugate JA, Dupras SK (2007). Cardiomyocytes derived from human embryonic stem cells in pro-survival factors enhance function of infarcted rat hearts. Nat Biotechnol.

[18] Akyurekli C, Le Y, Richardson RB, Fergusson D, Tay J, Allan DS (2015). A systematic review of preclinical studies on the therapeutic potential of mesenchymal stromal cell-derived microvesicles. Stem Cell Rev.

