# Supplementary material for: Bone marrow mesenchymal stem cell-derived endothelial cells increase capillary density and accelerate angiogenesis in mouse hindlimb ischemia model
Source: Stem Cell Res Ther. 2020 Jun 8;11:221. doi: 10.1186/s13287-020-01710-x (PMC7278145; doi:10.1186/s13287-020-01710-x)
Supplement: Supplementary file 1 — Additional file 1. Induction of unilateral mouse hindlimb ischemia model. [file 13287_2020_1710_MOESM1_ESM.docx]

Supplement

Induction of unilateral mouse hindlimb ischemia model. (1) Place the animal in the supine position after anesthesia and apply hair remove cream to thoroughly remove hair. (2) Place the animal under a surgical microscope. (3) Make an incision of the skin and open the wound more to expose the lower extremity vasculature. (4) Dissect and separate the femoral artery from groin to knee and make double knots separately. (5) Excise the full length of the femoral artery between the distal and proximal knots. (6) The excised femoral artery during operation.
