# Supplementary figures and images for: Bone marrow mesenchymal stem cell-derived endothelial cells increase capillary density and accelerate angiogenesis in mouse hindlimb ischemia model
Source: Stem Cell Res Ther. 2020 Jun 8;11:221. doi: 10.1186/s13287-020-01710-x (PMC7278145; doi:10.1186/s13287-020-01710-x)

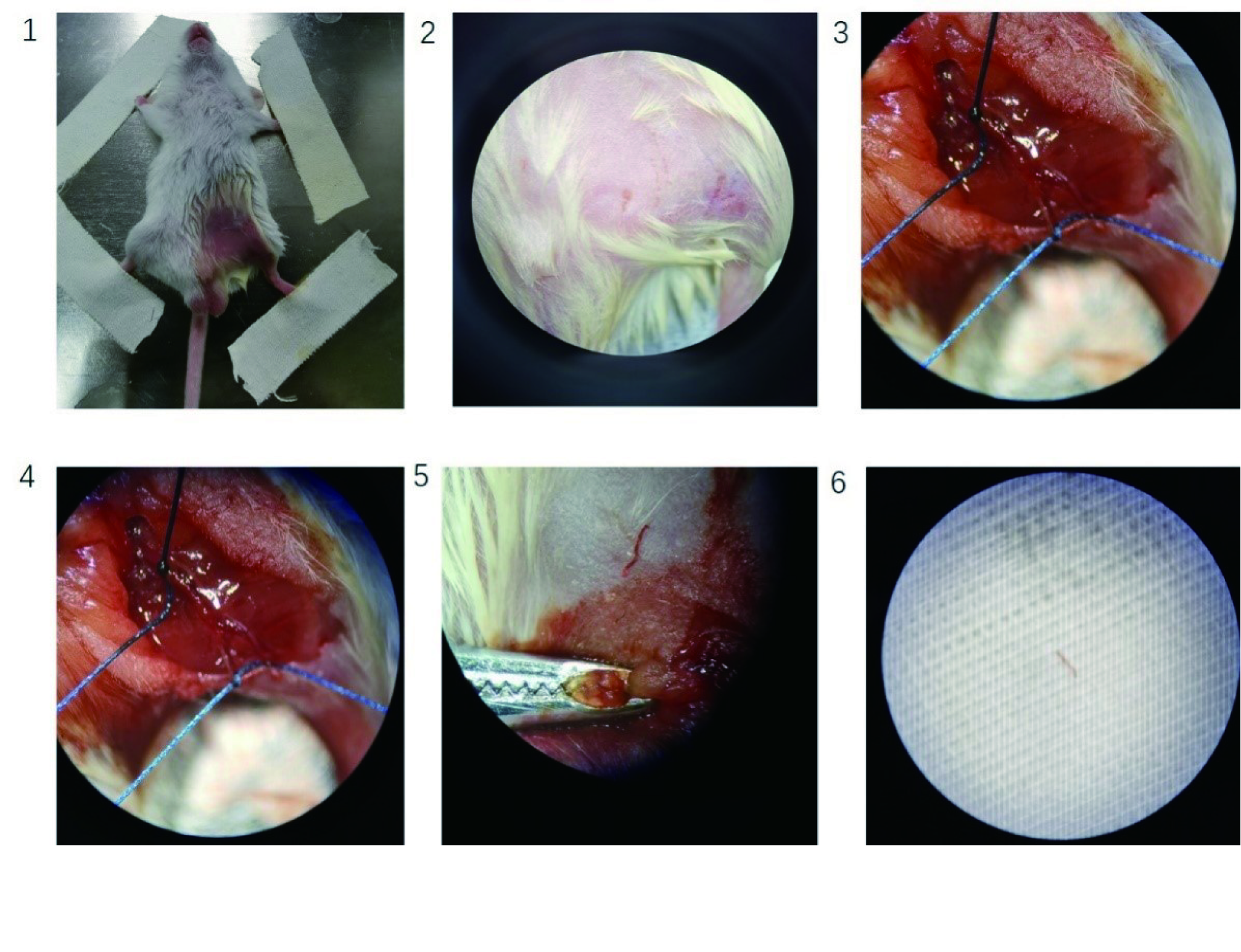

Supplement: Supplementary file 2 — Additional file 2. Supplementary figure. [file 13287_2020_1710_MOESM2_ESM.tif]
